# An Aggressive Form of Medullary Thyroid Carcinoma-Melanocytic Subtype: A Case Report

**DOI:** 10.7759/cureus.50310

**Published:** 2023-12-11

**Authors:** Dhaval Trivedi, Liziamma George

**Affiliations:** 1 Internal Medicine, NewYork-Presbyterian Brooklyn Methodist Hospital, Brooklyn, USA; 2 Pulmonary and Critical Care Medicine, NewYork-Presbyterian Brooklyn Methodist Hospital, Brooklyn, USA

**Keywords:** metastatic malignancy, melan a, medullary thyroid carcinoma, thyroid pathology, head and neck oncology

## Abstract

Medullary thyroid cancer (MTC) represents a small proportion of thyroid cancers. In MTC, melanin production is extremely uncommon. Few case reports have documented this rare variant, and follow-up on these cases has been very limited. Our case studies a 51-year-old female who initially presented with goiter. This tumor recurred multiple times despite surgery with rapid growth and poor response to radiotherapy. Microscopic examination showed high-grade malignant neoplasm with lymphocytic differentiation. Immunohistochemical studies were diffusely positive for S100, SOX10, and Melan-A. Histology confirmed melanocytic medullary carcinoma that had undergone a high-grade transformation with loss of epithelial and neuroendocrine expression. Due to the scarcity and rarity of this subtype, further evaluation and case studies are needed for further categorization and prognostication.

## Introduction

Medullary thyroid cancer (MTC) is a tumor that originates from the parafollicular cells (C cells) within the thyroid gland. The elevated production of calcitonin is characteristic of MTC and serves as an essential marker for the diagnosis of this neoplasm [1]. Although MTC is the third most common thyroid malignancy, its exact incidence remains unknown; the incidence peak is between the fourth and fifth decades with a wide range of age on presentation. The prevalence of MTC is approximately 3-5% of all thyroid malignancies; no difference in gender discrimination has been reported. MTC comprises two subtypes, namely, sporadic, accounting for 75% of cases, and familial, which constitutes the remaining 25% [2]. The most significant prognostic factors in MTC include calcitonin levels and doubling time. However, recent studies have also explored the use of serum carbohydrate antigen 19-9 positivity as a prognostic indicator in advanced MTC [3]. Thyroid ultrasound remains the primary method for initial diagnostic planning, with limited applicability of computed tomography (CT), magnetic resonance imaging (MRI), and positron emission tomography (PET) imaging. As complete surgical resection represents the definitive treatment for MTC, early diagnosis remains a crucial challenge.

Genetic testing has been performed as the RET (rearranged during transfection) mutation in the neural crest tissue in the thyroid gland can lead to MTC; germline mutations are associated with MEN2 and familial MTC. Notably, 40-50% of sporadic MTCs have acquired RET protooncogene mutation. RET encodes for receptor tyrosine kinase, which is expressed in neural cells and neuroendocrine thyroid cells, such as parafollicular cells. RET is a versatile kinase capable of directly phosphorylating multiple downstream targets. These targets include, but are not limited to, furthering cell proliferation and differentiation, promoting neuronal survival through focal adhesion kinase regulation, influencing tumor formation and metastasis, activating protein kinase C enzymes, regulating intracellular transport, initiating PI3K/AKT activation, and inducing RAS/ERK and MAPK pathways. These pathways collectively contribute to cellular differentiation and proliferation [4].

Therapeutically targeting RET oncogenes in MTC has shown promise, particularly with the advent of targeted therapies [5]. Family history, early intervention, and serum diagnostic testing and analysis remain pivotal for achieving early diagnosis.

Melanocytic MTC is morphologically distinguished by the presence of melanin deposition within the cytoplasm of the tumor cells and is an exceedingly rare phenomenon, with only a few case reports documenting such occurrences [6-18]. Given the extreme rarity of this presentation, further documentation and comprehensive analysis are necessary to gain a deeper understanding of this subtype.

## Case presentation

A 52-year-old female with a medical history encompassing allergic rhinitis, anemia, hypertension, latent syphilis, hyperlipidemia, obesity, pre-diabetes, and fibrocystic breast disease sought consultation with her endocrinologist due to a multinodular goiter. There was no family history of thyroid-related conditions. A thyroid ultrasound was performed to assess her goiter which revealed a right-sided nodule exceeding 5 cm in size prompting a referral for a biopsy. Physical examination was negative for signs of tracheal deviation, thyromegaly, or voice alterations. Laboratory results obtained at the time of biopsy were noteworthy for elevated levels of chromogranin A, calcitonin, and carcinoembryonic antigen (CEA), while levels of thyroid-stimulating hormone and free T4 remained within the normal range (see Table 1).

**Table 1:** Initial labs on presentation.

Lab study	Lab value	Reference range
Chromogranin A	123.2	0.0–101.8 ng/mL
Calcitonin, serum	8,643	0.0–5.0 pg/mL
Carcinoembryonic antigen	86.2	0.0–3.0 ng/mL
Thyroid-stimulating hormone	1.53	0.4–4.2 µIU/mL
Free T4	1.22	0.93–1.70 ng/dL
Calcium	9.5	8.5–10.5 mg/dL

Fine-needle aspiration was conducted twice, and pathological examination confirmed the presence of melanocytic subtype MTC. Immunostaining was positive for the following tumor markers: melan-A, calcitonin, CEA, synaptophysin, AE1/AE3, and CAM5.2. Following this immunohistological diagnosis, consultation with the otorhinolaryngology team led to a planned total thyroidectomy.

Histopathology of the total thyroidectomy specimen revealed a 6 cm encapsulated carcinoma with no signs of angiolymphatic invasion, consistent with a T3aN0a stage (with a mitotic rate of 10) MTC of the melanocytic variant. Notably, tumor markers tested positive for AE1, AE3, CK7, synaptophysin, INSM1, CEA, TTF1, and HMB45 (with focal positivity). Laboratory studies conducted at two and six-week intervals post-procedure are shown in Table 2.

**Table 2:** Labs post-thyroidectomy at the two and six-week follow-ups.

Lab study	Lab value two weeks post-surgery	Lab value six weeks post-surgery	Reference range
Calcitonin, serum	12.1	<2	0.0–5.0 pg/mL
Thyroid-stimulating hormone	0.185	0.01	0.4–4.2 µIU/mL
Free T4	Not performed	1.8	0.93–1.70 ng/dL
Calcium, serum	Not performed	9.8	8.5–10.5 mg/dL

At the two-month follow-up post-total thyroidectomy, bilateral neck adenopathy was detected on physical examination. A fine needle biopsy was performed, and the findings indicated a neoplasm with positive tumor markers for SOX100 and S100. The patient progressively developed dysphagia with neck discomfort and swelling and presented to the emergency department for evaluation. Upon video laryngoscopy, a hypomobile left vocal fold and a palpable firm thyroid mass were noted. CT imaging revealed several significant findings, including a new soft tissue density at the site of the previous thyroidectomy, focal narrowing of the trachea at the surgical site with normal patency distally, and the presence of a new 1.9 cm × 2.6 cm lymph node in the anterior mediastinum.

As a result of these findings, the patient underwent an open biopsy along with concurrent debulking of the thyroid tumor in the operating room. Pathological examination of the resected tissue revealed a high-grade malignant neoplasm with melanocytic differentiation involving skeletal muscle and associated soft tissues, along with invasion into both small and large-caliber blood vessels. Tumor markers were found to be positive for S100, SOX10, and melan-A. Molecular testing for the *BRAF p.V600* mutation was negative. Serial CT imaging of the neck demonstrated a 5.7 cm heterogeneous fluid collection in the thyroid surgical bed, consistent with postoperative changes. A physical examination indicated that the prior incision was clean, dry, and intact, with firm nodularity at the bilateral ends of the incision, which remained stable, and the patient was safely discharged.

Despite being discharged, the patient returned for re-evaluation the following day due to experiencing dyspnea. Upon a repeat examination, otorhinolaryngology fibroscopy detected a mass along the left lateral pharynx, measuring approximately 1 cm in its largest diameter. Importantly, this mass was not causing any airway obstruction. A subsequent CT scan of the neck confirmed the presence of a postoperative 5.1 cm heterogeneous fluid collection, consistent with previous findings. Additionally, the CT scan revealed an enlarged superior mediastinal lymph node, measuring 4.1 cm × 2.9 cm, with evidence of invasion into the anterior strap muscles.

To address the patient’s condition, intravenous dexamethasone was initiated at a dose of 6 mg every eight hours, which was subsequently tapered over a five-day course. Following this treatment, the patient was discharged with plans for a follow-up appointment with radiation oncology to begin radiotherapy for managing this rapidly progressing recurrence.

**Figure 1:**
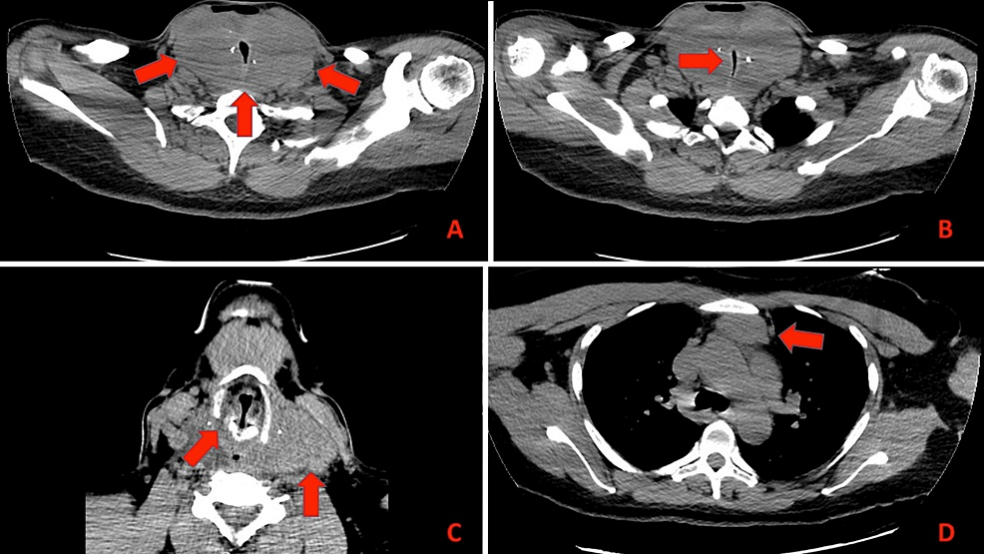
Computed tomography of the neck without contrast. (A) Large homogeneous lobulated soft tissue mass within the region of the thyroidectomy bed measuring approximately 6.5 x 12.2 x 6.2 cm. (B) Pronounced circumferential narrowing of the lower cervical and upper thoracic trachea. (C) Mass extends anteriorly and superiorly into the hypopharynx where there is ill-defined soft tissue within the right true vocal cord. (D) Mass extends inferiorly into the superior mediastinum where there is also a nodal agglomeration measuring 2.4 x 4.4 cm.

Upon the initiation of radiotherapy, both physical examination and advanced imaging uncovered compelling evidence of multiple solid and immobile tumors in the region where the thyroid bed was situated. The largest of these tumors exerted pressure on the carotid sheath, causing it to shift toward the posterior triangle of the neck. Additionally, another distinct nodule was observed in the lower part of the neck, occupying the space where the isthmus had previously been located. Furthermore, a relatively sizable mass measuring 4 cm was identified within the left thyroid bed. Most notably, there was an approximate 90% circumferential constriction of the airway, with invasion into critical structures such as the larynx, right vocal fold, and left piriform sinus (see Figures 1A-1D).

The patient presented with mild stridor and a raspy voice. Immediate simulation for radiation therapy with intravenous contrast revealed substantial soft tissue masses that were encasing the trachea, extending into the superior mediastinum, and causing a narrowing of the tracheal lumen.

Initially, the proposed treatment plan was to initiate a twice-daily fractionation schedule to counteract repopulation. However, treatment posed challenges due to respiratory distress and the patient’s claustrophobia while on the radiation table. As a result, the first fraction, totaling 148.5 cGy out of the planned 200 cGy, was administered in the morning, and the remaining dose of 348.5 cGy was given in the evening. Following the first day of treatment, the patient developed stridor and received decadron, racemic epinephrine, and HeliOx treatment.

Due to her worsening symptoms, otorhinolaryngology recommended and performed an urgent fiberoptic intubation in the operating room. Following this intervention, the radiation oncology fractionation regimen was revised, and treatment was resumed while the patient remained intubated, with sessions now conducted once daily. Subsequently, the patient was successfully extubated, and there was notable shrinkage of her thyroid mass.

Subsequent radiation treatments were administered without significant side effects or interruptions, and the patient was discharged with plans for outpatient follow-up. In total, the patient received a cumulative radiation dose of 5,885.5 cGy, with the initial three-dimensional conformal radiation therapy (3DCRT) technique utilized for the first five fractions, followed by intensity-modulated radiation therapy (IMRT) for the remaining 21 fractions.

Following the radiotherapy, a PET/CT scan was conducted due to the onset of significant right pelvic pain. The scan revealed extensive bony metastasis, which can be visualized in Videos 1-3. In response, immediate palliative radiotherapy was administered to address the metastasis in the right hemipelvis. The patient’s respiratory symptoms worsened, leading to the development of pleural effusion. She required continuous bilevel-positive airway pressure for support. During this time, there were extensive discussions regarding the goals of care, and the patient made the decision to forego intubation if her condition deteriorated further. Unfortunately, the patient’s condition deteriorated significantly during her hospitalization, resulting in worsening hypoxia, and she eventually passed away.

**Video 1:** Axial fused positron emission tomography-computed tomography scan showing extensive metastasis.

**Video 2:** Coronal fused positron emission tomography-computed tomography showing extensive metastasis.

**Video 3:** Three-dimensional maximum intensity projection positron emission tomography-computed tomography image showing extensive metastasis.

## Discussion

MTC is an uncommon type of thyroid malignancy that originates from perifollicular cells. The typical presentation of MTC is known to involve multidirectional differentiation, and tumor cells can produce a wide range of hormonal and non-hormonal products, including mucin, peptides, and amines [19]. Nevertheless, melanin production within MTC has been rarely reported or documented in the medical literature.

The phenomenon of melanin production in MTC was initially described by Marcus et al. [12] in 1982. Since then, approximately 13 cases have been reported in the literature, excluding cases from our institution. Intriguingly, all these reported cases exhibited diverse histomorphological features.

Due to the scarcity of this specific subtype, the prognosis and mortality associated with it remain unclear. It is worth noting that prior cases with high mitotic counts experienced recurrent disease, particularly in the soft tissues of the neck, and developed bone metastases within two years following surgery [20].

Our case involves a patient diagnosed with the MTC-melanocytic subtype, who underwent total thyroidectomy. Unfortunately, aggressive recurrence followed. Immunohistochemical analysis revealed positive results for melan-A, calcitonin, CEA, synaptophysin, AE1/AE3, and CAM5.2, correlating with MTC [5,6]. Post-procedurally, the patient initially responded well, with improved calcitonin levels and normal thyroid levels. However, during follow-up visits, progressive adenopathy was observed, leading to a biopsy. The biopsy results indicated tumor expression of SOX100 and S100, commonly used in evaluating neural crest-derived tumors [19]. The initial biopsy also showed the presence of melan-A and HMB45, consistent with prior cases of the MTC-melanocytic subtype. Notably, melan-A exhibited a significant association with worsening metastasis in our patient, unlike HMB45 [5].

In the planning of radiation oncology decision-making, the initial proposal for radiation therapy included a twice-daily fractionation schedule to counteract tumor repopulation. Dosing was calculated using biologically effective dose and equivalent dose calculators, assuming an alpha/beta ratio of 10. However, during the first radiation treatment and the development of worsening respiration, only 148.5 cGy of the planned 200 cGy was delivered in the morning, with the full dose (348.5 cGy) given in the evening. Subsequently, she experienced stridor and underwent successful fiberoptic intubation. The treatment plan was revised, and she continued with once-daily treatments. Seventeen days after the initial therapy, she was eventually extubated. Due to shrinkage in her thyroid mass, a repeat CT simulation was performed. Treatment resumed, and she completed the course without major issues, receiving a total of 5,885.5 cGy. The therapy involved 200 cGy and 267 cGy daily fractions to the thyroid mass, initially using the 3DCRT technique for the first five fractions, followed by IMRT for the remaining 21 fractions. There was one break for re-simulation due to a significant treatment response.

IMRT was indicated because steep dose gradients outside the target must be achieved to avoid exceeding the tolerance dose to critical structures in close proximity. A decrease in the amount of dose inhomogeneity is required to prevent an excessive dose hotspot within the treated volume, thereby avoiding excessive early or late normal tissue toxicity. Although this radiation therapy showed a significant response in tumor burden, she progressively experienced worsening metastasis and clinical decline.

In the utilization of alternative treatment modalities, therapy targeting the RET protooncogene has been employed for therapeutic purposes in MTC. Studies from the early 1990s demonstrated that point mutations in the RET protooncogene, located on chromosome 10q11.2, increased the risk of MEN2A, MEN2B, and familial MTC. While there is a consensus on the need for prophylactic thyroidectomy in families with hereditary MTC, the timing of this procedure is contingent upon the site and associated risk of the RET codon mutation. Current investigations are centered around identifying molecular targeted therapies that selectively inhibit kinase receptors such as VEGFR2, VEGFR3, and EGFR/HER1, utilizing anilinoquinazoline analogs such as vandetanib. These studies have revealed significant inhibitory effects of these targeted therapies on the growth of thyroid cancer cell lines featuring spontaneous RET rearrangements. Additionally, findings indicate that 20% of patients undergoing these targeted therapies experience partial remission. Post-treatment, there was a notable reduction in serum levels of calcitonin and CEA [5].

The case currently under discussion exhibited a high mitotic count, with recurrence occurring within three months of surgical intervention and eventual mortality within eight months of diagnosis. Whether the presence of melanin contributes to the severity of disease progression remains uncertain due to the rarity of this condition. Comprehensive analysis and further investigation are warranted to ascertain and comprehend the aggressiveness of this particular subtype.

## Conclusions

The melanocytic variant of MTC is exceptionally rare and scientifically intriguing. Its potential for heightened aggressiveness, deviating from the typically indolent nature of conventional MTC, poses significant clinical challenges, potentially leading to rapid progression, increased metastasis rates, and distinct molecular profiles. However, the current scientific understanding of this variant remains limited, necessitating extensive research efforts, including large patient cohorts, to reveal its characteristics, predictive factors, and potential therapeutic strategies.

Achieving a comprehensive understanding of this variant’s characteristics and clinical behavior is crucial, demanding the accumulation of a sufficiently large patient cohort to enable statistical analyses and pattern recognition. Such studies may uncover commonalities and distinctions, pinpoint predictive factors, and offer insights into therapeutic strategies. Furthermore, delving deeper into the molecular and genetic underpinnings of the melanocytic variant holds promise for elucidating the mechanisms behind its heightened aggressiveness, potentially revolutionizing treatment approaches for this specific subtype.

Unfortunately, the realm of clinical trials investigating molecular targeted therapies in advanced thyroid cancer faces a notable scarcity of studies. The limitation in obtaining sufficient and relevant tissue samples has impeded the robust development of clinical trials exploring the potential efficacy and safety profiles of molecular targeted therapies for advanced thyroid cancer. The gathering of case studies akin to ours is essential for enhancing our understanding and establishing a robust foundation to further advance targeted therapies.

This dearth of comprehensive studies underscores the critical need for increased efforts and resources dedicated to overcoming these challenges. Addressing these gaps is essential to advancing our understanding and clinical strategies in the realm of advanced thyroid malignancies. By fostering a more comprehensive research landscape, we can unlock potential breakthroughs that may redefine the therapeutic landscape for thyroid cancer, including novel approaches and targeted interventions tailored to specific variants, such as the melanocytic variant within MTC.
